# Flow-Diversion Panacea or Poison?

**DOI:** 10.3389/fneur.2014.00021

**Published:** 2014-02-28

**Authors:** Mario Zanaty, Nohra Chalouhi, Stavropoula I. Tjoumakaris, Robert H. Rosenwasser, L. Fernando Gonzalez, Pascal Jabbour

**Affiliations:** ^1^Department of Neurosurgery, Jefferson Hospital for Neuroscience, Thomas Jefferson University, Philadelphia, PA, USA

**Keywords:** flow diversion, pipeline, intracranial aneurysm, FRED, Surpass, PED

## Abstract

Endovascular therapy is now the treatment of choice for intracranial aneurysms (IAs) for its efficacy and safety profile. The use of flow diversion (FD) has recently expanded to cover many types of IAs in various locations. Some institutions even attempt FD as first line treatment for unruptured IAs. The most widely used devices are the pipeline embolization device (PED), the SILK flow diverter (SFD), the flow redirection endoluminal device (FRED), and Surpass. Many questions were raised regarding the long-term complications, the optimal regimen of dual antiplatelet therapy, and the durability of treatment effect. We reviewed the literature to address these questions as well as other concerns on FD when treating IAs.

## Introduction

Endovascular therapy is now the treatment of choice for intracranial aneurysms (IAs) for its efficacy and safety profile. Still, many aneurysms such as large, giant, wide-necked, and fusiform aneurysms are considered more challenging and less amenable to traditional endovascular coiling (1). Stent-assisted coiling (SAC) and balloon-assisted coiling (BAC) were alternative techniques developed to deal with such complex aneurysms, but studies have shown their less than expected efficacy given their high rate of recanalization (2–5). The flow-diversion (FD) technique has brought a feasible and effective solution. In addition, the use of FD has recently expanded to cover many types of IAs in various locations. Some institutions even attempt FD as first line treatment for unruptured IAs. The most widely used devices are the pipeline embolization device (PED), the SILK flow diverter (SFD), the flow redirection endoluminal device (FRED), and Surpass. Many questions were raised regarding the long-term complications (i.e., delayed bleeding and device migration), the optimal regimen of dual antiplatelet therapy (APT), and the durability of treatment effect. We reviewed the literature to address these questions as well as other concerns on FD when treating IAs.

## Flow-Diversion Method

The FD technique relies on a concept of endoluminal reconstruction of the parent artery and the aneurysm neck by excluding the aneurysm from the circulation. The stasis of blood flow in the aneurysm leads to an inflammatory response followed by thrombosis and “healing” of the aneurysm while the stent acts as a scaffold for neointimal proliferation and remodeling of the parent vessel. Therefore, the FD approach is considered physiologic as it restores the normal homeostasis. A recent study showed that flow-diverter device (FDD) reduces the velocity in the aneurysm sac significantly more than multiple “non-flow diverter” stents, even though both dramatically reduce the aneurysmal fluid movement (6). To break the communication between the parent artery and the aneurysm while maintaining a patency of sidewall branches, the device must fulfill two requirements: a low porosity (metal-free to metal-covered area) and a high pore density (number of pores per square millimeters for a given porosity) (7, 8). However, sidewall branch occlusions do not always lead to ischemia since collaterals may maintain flow to the dependent area. Even more, when collaterals are not present, the increased demand for tissue perfusion may, in some cases, generate a pressure gradient sufficient to maintain an anterograde flow through the device (7).

The technique involves navigating an FDD through the arterial system and deploying it across the aneurysm neck. Proper deployment is essential as inadequate wall apposition may decrease the flow with consequent thrombus formation at the interface followed by thromboembolic events (8). Proper deployment and adequate wall apposition can be achieved by balloon (Boston angioplasty (9), though not always needed. More so, the increased turbulence along with the lytic enzymes released from platelet aggregation predisposes to a possible lysis of the aneurysmal wall that can usually occur in the following days post-op (10). This may lead to rupture and SAH if the aneurysm is not completely thrombosed. After stent deployment, there are no data-driven guidelines on optimal APT. Most of the time, the patient is maintained on dual APT for 3–6 months followed by lifelong monotherapy. In practice, the indication varies depending on the aneurysm location (anterior vs. posterior), and parent artery/side vessels stenosis (1, 8). The blood-thinning component makes FDD of limited use in ruptured aneurysm, at least not before the aneurysm is entirely secured. The heterogeneity of the response to APT, especially with clopidogrel, could explain the in-stent thrombosis on one hand, and the hemorrhagic events on the other. One study led by Lee et al. showed that all cases of intraprocedural thrombosis occurred in patients with poor response to antiplatelet treatment (11). Delgado et al. found that pre-procedure P2Y12 reaction unit (PRU) value of <60 (over-inhibition) was an independent predictor of perioperative hemorrhagic events and a PRU value of >240 (under-inhibition) was an independent predictor of perioperative ischemic events (12). Both a technically difficult procedure and labile hypertension are independent risk factors of thromboembolic and hemorrhagic complications. In practice, some authors recommend loading the patient 10 days prior to the procedure with 75 mg/day of clopidogrel (or another thienopyridine) and 81 mg/day of aspirin until 30–90% P2Y_12_ inhibition is achieved (9). In our institution, Clopidogrel assays are checked at baseline before the administration of Clopidogrel and then again just before the procedure. The percentage of inhibition is calculated and the dosage is adjusted to achieve a platelet inhibition between 30 and 90% before the procedure. Patients with resistance to Clopidogrel are switched to Prasugrel. Dual APT is envisioned for at least 6 months, followed by lifelong monotherapy of aspirin (81 mg).

**Figure 1:**
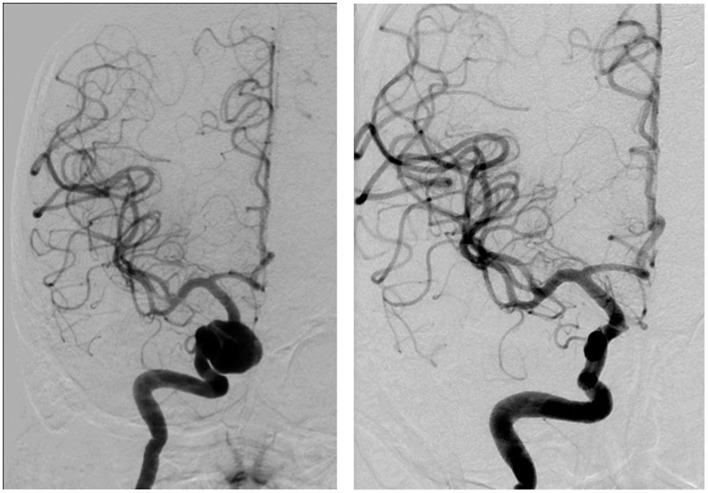
**Case 1**.

**Figure 2:**
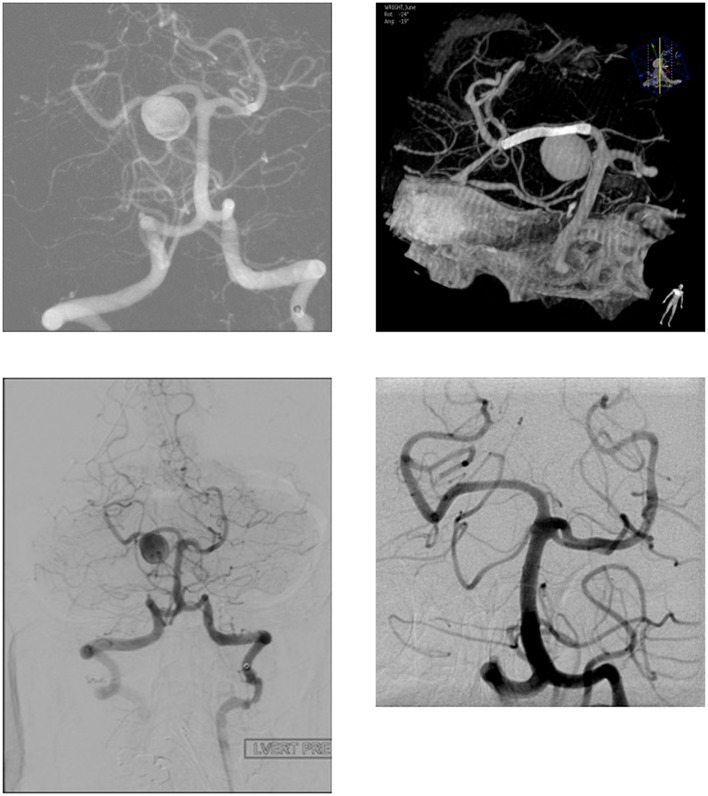
**Case 2**.

**Figure 3:**
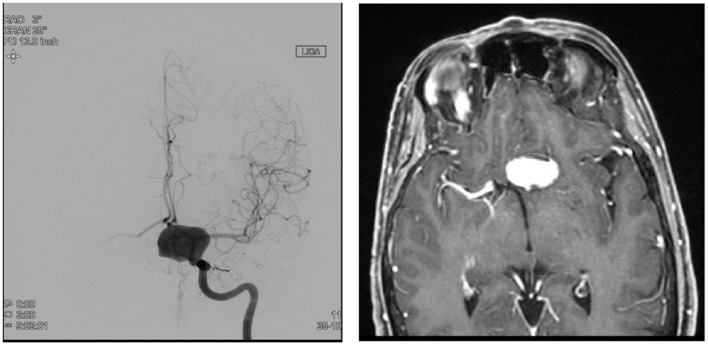
**Case 3**.

**Figure 4:**
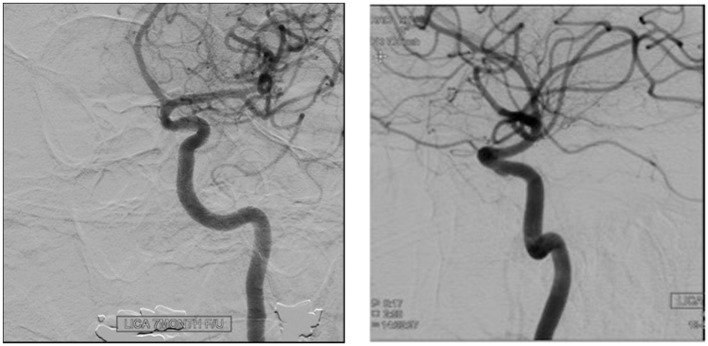
**Case 4**.

**Figure 5:**
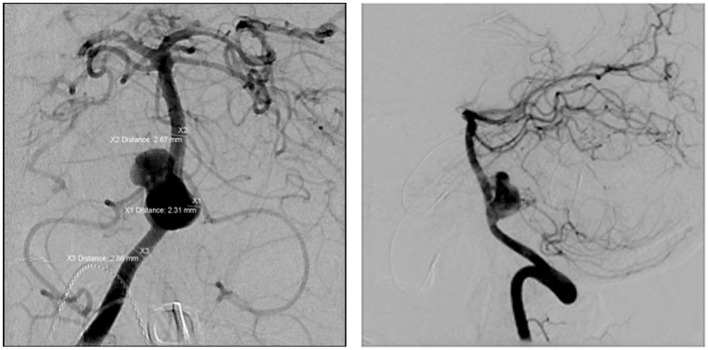
**Case 5**.

**Figure 6:**
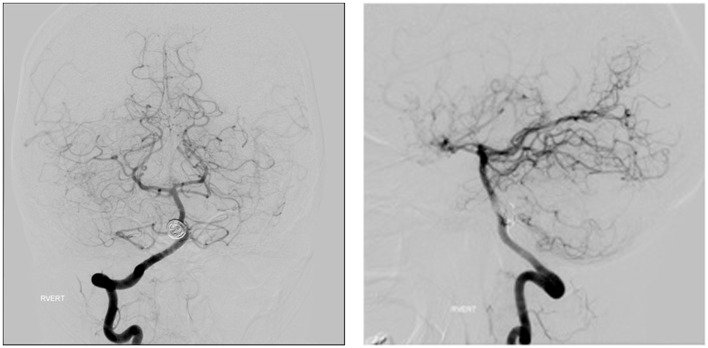
**Case 6**.

The FDDs offer the advantage of avoiding IA manipulation that increases the rupture risk as well as avoiding any coil insertion that may worsen the preexisting mass effect. The findings of Lylyk et al. (13) and Szikora et al. (14) were consistent with this advantage as they reported that improvement of mass effect symptoms occurred after FD treatment. On the contrary, the inflammatory changes inside the aneurysm may cause a transient worsening of the mass effect that can be seen initially after the procedure, by increasing the aneurysm size or perhaps by direct spread of the inflammation to the surrounding parenchyma (15). A well-known yet poorly understood complication is the rupture of previously silent IAs. One reason could be the hemodynamic alteration of flow after PED or SFD placement (16). Delayed hemorrhage is another unfavorable outcome whose risk factors are not fully elucidated. Evidence shows that large size, complex geometry, and high aspect ratio (>1.6) predispose to delayed hemorrhage (17). More so, Kuzmik et al. highlighted the unpredictability of FD by showing that even when the morphology and the location are similar, the treatment outcomes may differ enormously (18). One final major area of ambiguity is the delayed remote intracerebral hemorrhage (ICH), explained by some as shower emboli with hemorrhagic conversion and by others as a damping effect after FD that increases the pulsatility of distal vasculature and leads to small arteriolar rupture (15). As for the indications of FD (Table 1), it was used traditionally for large and giant aneurysms (diameter >10 mm), wide-neck aneurysms (neck width >4 mm), and aneurysms with a morphology unsuitable for coiling (fusiform and dissecting). In theory, FD can be used for any type of aneurysm but concerns remains about its use in bifurcation-aneurysms and whether it is worth using in small saccular aneurysms with low recurrence rate after coiling (15) (for illustrative pictures of aneurysm treated with PED, check Figures 1–6).

**Table 1:** **Indications and concerns regarding flow diversion treatment**.

Indications for flow diversion	Concerns
Diameter >10 mm	Bifurcation aneurysm
Neck width >4 mm	Small saccular aneurysm with low recurrence risk after coiling
Complex morphology: fusiform, dissecting	
Recurrence after coiling	

## Pipeline Embolization Device

The PED (ev3, Irvine, CA, USA) is a microcatheter-delivered, self-expanding, cylindrical stent composed of a mesh of 48 individual cobalt chromium and platinum strands. It has a low porosity, high metal coverage, and is specifically designed for FD. It is available in lengths up to 35 mm with diameters of 2.5–5 mm in 0.25 mm increments.

This device once initially used for large and giant aneurysm has been increasingly used for smaller and less complex ones. Recently, PED has been shown in the treatment of large (>10 mm) saccular unruptured aneurysms (UAs) to achieve higher occlusion rate, fewer recanalization rate, and similar morbidity and mortality than with traditional coiling (9). Another benefit of PED when compared to coiling is the fewer cost when the aneurysm is >0.9 cm^3^ and only one device is used (19). It is of highly importance to estimate the number of PED required as it affects the cost, the safety, and the efficacy of the procedure (20).

In a systemic review of the literature involving 10 studies, Leung et al. managed to pull out data on 414 patients with 448 IAs treated with PED (1) (Table 2). The mean number of PED used was 2 devices per IAs. The procedure-related complications were IAs rupture, ischemic strokes, non-IA-related intracranial hemorrhages, worsening of mass effect, and femoral/retroperitoneal hematomas. The overall symptomatic complication rate was 10.3% (46/447), of which 6.3% were exclusively intracranial vascular events (ischemic or hemorrhagic). The procedure-related mortality was 2.2% (9/413), mostly due to rebleeding. The morbidity and mortality rate following treatment of UAs was much lower than those following the treatment of ruptured ones (6.1 vs. 18.8%, 0.8 vs. 12.5%, respectively), but no statistical test was applied in their study. The limited number of ruptured IAs in the studies makes the difference in morbidity and mortality rates less valid. Still, the authors advised against the use of PED in context of ruptured IAs given the lack of evidence on its efficacy and safety in the literature. The authors found that IAs that have been previously stented posed a challenge to PED deployment, had a higher rate of vascular complications and a lower rate of complete obliteration. Thus, the previous findings should be taken into consideration while planning PED treatment for previously stented aneurysm. Complete obliteration was achieved in 82.8% (293/354) at 6-month follow-up, which compares favorably with SAC (21) and balloon-assisted embolization (22). However, a more scientific comparison is needed before concluding. Fargen et al. (16), in their review of reported complications associated with the PED, found similar morbidity (5.3%) and mortality (1.3%) rates.

**Table 2:** **Morbidity, mortality, and occlusion rates for FDDs as reported from case series, systemic reviews, and meta-analysis**.

	FDD used	Morbidity rate (%)	Mortality rate (%)	Complete occlusion at follow-up (%)
Leung et al. (systemic review)	PED (1–3.2/patient)	Ruptured and unruptured aneurysms: 6.3	Ruptured and unruptured aneurysms: 2.2	82.8
		Ruptured only: 18.8	Ruptured only: 12.5	
		Unruptured only: 6.1	Unruptured only: 0.8	
Saatci et al.	PED (1.3/patient)	1	0.5	91.2
Brinjikji et al. (meta-analysis)	PED and SFD	5	4	76
Pistocchi et al.	PED and SFD	3.7	0	78.9
Briganti et al. (meta-analysis)	PED and SFD	3.7	5.9	85

In another study, PED was used to treat complex, simple, wide-necked, giant, small, fusiform, dissecting, and saccular aneurysms (23). Technical deployment was successful in all cases. On average, the number of PED device used was 1.91 per aneurysm (23). Reported symptomatic complications constituted 13.9% and included thromboemboli, ICH, dissection, and death. Multiple stents were used to ensure proper coverage and care was taken not to cover the perforator with more than one stent.

Piano et al. managed to treat successfully 47 aneurysms with Silk and 57 with PED without any technical failure (24). The morbidity and mortality rate, including delayed complications were both 3%. Follow-up after 6 months showed complete occlusion in 85% of the cases. At 1-year follow-up, no recanalization was observed.

On the other hand, some authors reported remarkably lower rates of mortality and morbidity. Saatci et al. treated 251 aneurysms in 191 patients using PED with a morbidity rate of 1% and a mortality rate of 0.5%. Similarly, Pistocchi et al. in a series of 30 aneurysms beyond the circle of Willis reported no mortality and a morbidity rate of 3.7% (25). Finally, Brinjikji et al. in their meta-analysis of 29 studies, examined 1452 patients with 1654 IAs and found that the procedure-related morbidity and mortality with FDDs (both PED and SFD) were 5% (95% CI; 4–7%) and 4% (95% CI; 3–6%), respectively, and the complete occlusion rate to be 76% (95% CI; 70–81%) (26) (Table 2). They also noted that treatment of posterior circulation aneurysm is more prone to ischemic events, particularly perforator infarction when multiple devices are used. Overall, the perforator occlusion risk was 3%.

Another major concern with PED stent is the higher risk of spontaneous migration, which could be early or delayed, and results in aneurysm rupture or ischemic events (27). It is best managed by placing additional stents to achieve once again complete coverage or even more precociously, by taking preventive measures in the first place, such as using longer PEDs, achieving complete expansion, avoiding dragging and stretching of the PED that distorts and shortens the device, and finally using adjunctive coiling to prevent any prolapse of the PED into the aneurysm (27).

## Silk Flow-Diverter

SILK stent (Balt Extrusion, Montmorency, France) is a self-expanding flexible stent constructed of woven nitinol strands and platinum microfilament with low porosity and high metal coverage of 35% (28). It is available in 2–5 mm diameters and in 15–40 mm lengths.

Treatment with Silk has been shown to be efficacious, safe, with reasonable morbidity and mortality. Silk was found in some studies to achieve similar occlusion rates to PED, but with the cost of higher early complications (25, 29). Mortality rates for the device reported from case series ranged from 0 to 8% and morbidity rates from 3.9 to 15% (29–33). Complete aneurysm occlusion rates were 50–69% at 6-months follow-up, and one study of 26 aneurysms reported an occlusion rate of 86% at 1 year (29). However, the number of studies does not allow a fair head-to-head comparison with PED.

A multicenter study enrolling 25 Italian centers evaluated 273 patients with 295 IAs that were treated with Silk or PED (10). The trial included fusiform, large, giant, and wide-necked aneurysms. Also, small aneurysms deemed untreatable by conventional coiling were included in the study. The morbidity and mortality rate in the anterior location were 2.3 and 3.5%, respectively. In the posterior location, the reported morbidity and mortality rate were 5.4 and 19%, respectively. The overall mortality rate was 5.9% and the morbidity rate was 3.7%. Hemorrhagic events occurred in 5.5% of patients, of which 50% were device-related complications: seven patients had delayed aneurysm rupture (two with SFD, five with PED), and one patient had middle cerebral artery (MCA) perforation during PED retrieval after distal migration of the device. The remaining seven had hemorrhagic events that were deemed procedure-related such as iatrogenic vessel perforation and ICH on APT.

Thromboembolic events occurred in 4.8% of patients and included: side-branch occlusions (one with Silk, two with PED), in-stent thrombosis (three with Silk, three with PED), and procedure-related ischemia. The authors also noted a higher mortality in the subgroup of intracavernous aneurysms (4%) treated by FDD and in the small subgroup of patients with giant complex aneurysm treated by coiling (35.7%; 5/14) (10). Thus, it is recommended that extradural aneurysm be treated only if symptomatic and in expert hands (10). Finally, failure of device deployment, device mispositioning, in-stent aggregation, and other technical complications occurred in 21.5% of the procedures without clinical manifestations. This high rate may be related to the recent introduction of the devices (10). At 3-month follow-up, complete occlusion was achieved in 85% of patients. The remaining 15% were exclusively aneurysms of the anterior circulation. Finally, the authors conducted a meta-analysis of six studies, including their own (13, 14, 30, 33, 34) that showed an overall morbidity rate of 6.2% (CI 95% 2.0–6.7%) and a mortality rate of 3.4% (CI 95% 2.4–4.7%). The safety and efficacy of FD use in bifurcation aneurysms remain unknown. So far, FD has been reserved for bifurcation aneurysms that are not amenable to surgery and when other means of endovascular treatment are deemed risky (35). A study was recently published on PED treatment of 25 aneurysms located at MCA bifurcation or M2 in case one of the bifurcating branches or a distal branch originated directly from the aneurysm sac (35). Follow-up (3–30 months) showed a complete occlusion rate of 84%. They had no mortality and an SAH as the only procedural complication. Even with the limited number of cases and the lack of long-term follow-up, the results are somewhat encouraging.

## Surpass Flow-Diverter

The Surpass flow diverter (Surpass; Stryker Neurovascular, Fremont, CA, USA) is a new device that comes in various diameters and length so that most of the time, one single stent is sufficient for aneurysm occlusion (8). The essential features of the device are a low porosity, and a uniformly distributed high pore density that remains constant regardless of the diameter. The advantage of a single device use is the maintenance of a constant porosity by alleviating the need of random telescoping of two implants. This offers additional protection for side branches by allowing a better control of porosity and pore density (8). In a study of 37 patients, harboring 49 UAs, a single device was used to treat each patient except in one case, where telescoping of two devices had to be done to cover the whole diseased segment of a giant fusiform basilar aneurysm (8). The study included: large, giant, wide-necked, dissecting/fusiform, and blister-type aneurysms. Recurrent or recanalized saccular aneurysms that have been previously coiled and small aneurysms that were judged to have a high risk of rupture were also included. In this study, 38 devices were used to treat 49 aneurysms, which means an average of 0.8 devices per aneurysm. There was no failure of device delivery. Complete occlusion was achieved in all 35-non-bifurcation aneurysms. The higher occlusion rate in the study could be due to the maintenance of pore density with the change in diameter of the device, in contrast to PED and Silk (8). As experimental studies have showed, a constant pore density over the length of the aneurysm neck leads to a more efficient FD and durable aneurysm occlusion (7). Still, this conclusion would be premature given the absence of control group in the study. Even more, the high proportion of small aneurysms included in this study could have influenced the higher occlusion rate and the lower complication rate (8). In comparison, none of the 14 bifurcation-aneurysm received complete neck coverage, and only 50% were occluded on follow-up after 6 months. As for the complications, a clot formed in one case over the Surpass stent and was successfully treated by intra-arterial abciximab. Other complications were: small asymptomatic MCA perforation, 2 internal carotid artery traumatic dissections by the microwire (one of which was noticed during the operation and successfully treated by Surpass). There was no major intraoperative vasospasm or migration of the implant, and no periprocedural mortality or significant morbidity. During follow-up, four patients (10.4%) experienced transient ischemic events and one patient (3%) developed a minor stroke 1 month after stopping clopidogrel with persistent neurological deficit. Therefore, the morbidity rate was comparable to coiling and SAC as well as to other FD devices such as PED and Silk (36, 37) (Table 3). The majority showed improvement or resolution of their symptoms while the remaining remained stable. The authors concluded that Surpass flow diverter is safe, reliable, and very effective given the right indications. The main limitations of this single trial are the small number of patients and the high proportion of small aneurysms (37).

**Table 3:** **Mortality, morbidity, and complete occlusion rate as reported from case series**.

Device	Mortality rate (%)	Morbidity rate (%)	Complete occlusion (%)
PED	0–6	0–9	76–91.2
SILK	0–8	3.9–15	69

## Flow Redirection Endoluminal Device System

The FRED system (MicroVention, Tustin, CA, USA) is a new generation of FDDs used in the treatment of IAs. Diaz et al. reported the first use of FRED in the western world, in their small trial of 13 patients with 14 IAs (38). They had no immediate complications or technical difficulties. However, the trial lacked follow-up on long-term complications and angiographic results. The authors viewed the ability of the device to maintain its internal shape when navigating in tortuous and kinky cerebral vessels as an improvement over the older generations of FDDs (38). The authors were encouraged by the outcomes of the study.

## Novel Use of Flow-Diversion Devices

Newly, PED was used for arterial deconstruction instead of reconstruction by inducing a progressive thrombosis of the parent artery that fed the aneurysm (39). In this case report, a patient with a giant distal MCA aneurysm who refused open surgery did not tolerate superselective balloon-occlusion test and catheterization of the aneurysm for PED treatment was unattainable. PED was used for a compromise between branch occlusion and FD. The patient eventually tolerated the progressive thrombosis promoted by PED. It was postulated that chronic ischemia favored the expression of vascular endothelial growth factor, thereby inducing the development of a collateral network by angiogenesis, and restoring the blood flow (39, 40). Nyberg et al. took advantage of the unique property adherent to PED in order to salvage the MCA after surgical clipping of the internal carotid artery aneurysm that left the flow compromised in the MCA (41). The final configuration of PED as well as the final radial forces are related to the material properties of PED and, unlike other FDD, are operator dependent such as the more foreshortened the device is, the greater its radial force (41). The diamond-like configuration of the strands provides PED with high resistance to crushing; more pressure is required to crush the device to its pre-deployment configuration then to deploy it (41). PED was used to expand the MCA against the clip, with the help eventually of balloon angioplasty.

Flow-diversion is also being used for intracranial dissecting aneurysms. In the acute phase, FD can be problematic. First, navigation of the device through tortuous vessel is challenging. More so, the patients have to be put under aggressive antiaggregation, which could be problematic in case of rerupture and rebleeding when the aneurysm is not completely secured. This risk of hemorrhage should be weighed against the risk of ischemic events. On the other hand, FD offers the advantages of avoiding aneurysm catheterization. In addition, the densely packed woven mesh slow or avert the progression of the dissecting aneurysm by holding the flap up against the wall and vessel remodeling (42). Therefore, FDDs are being used effectively and with caution in the treatment of acute dissecting aneurysms. Still, parent artery occlusion, when feasible, remains the preferred and safest treatment option (42, 43).

## Conclusion

Treatment of UAs with FDDs is safe and effective with high complete occlusion rates. The procedure-related morbidity and mortality varied in the literature, yet remained encouraging. Posterior circulation aneurysms, previously stented aneurysm, bifurcation-aneurysm, and multiple stent use may result in poorer outcomes. These factors must be thought of when determining the type of treatment. Careful manipulation of the device, proper device deployment, and complete coverage of the neck help reduce the procedure-related complications. The use of FD in recently ruptured IAs has not been solidly proven safe. More trials are needed to clarify the management of aneurysms that failed treatment and the management of clinical adverse outcomes as well as their prevention. Finally, the remarkable efficacy that led recently to PED use in smaller and less complex aneurysm should be challenged in randomized controlled trials with traditional endovascular coiling.

## Conflict of Interest Statement

The corresponding author, Pascal Jabbour, MD, is a consultant at Covidien and CNV. The other co-authors declare that the research was conducted in the absence of any commercial or financial relationships that could be construed as a potential conflict of interest.
